# Partial Recovery of Audiological, Vestibular, and Radiological Findings following Spontaneous Intralabyrinthine Haemorrhage

**DOI:** 10.1155/2013/941530

**Published:** 2013-12-24

**Authors:** Thomas Pézier, Krisztina Baráth, Stefan Hegemann

**Affiliations:** ^1^Interdisciplinary Centre for Vertigo and Balance Disorders, ORL Klinik, University Hospital Zurich, Frauenklinikstrasse 24, 8091 Zurich, Switzerland; ^2^Medical Radiological Institute Zurich, Toblerstrasse 51, 8044 Zurich, Switzerland

## Abstract

The diagnosis, work-up, and treatment of sudden sensorineural hearing loss and sudden vestibular loss vary widely between units. With the increasing access to both magnetic resonance imaging and objective vestibular testing, our understanding of the various aetiologies at hand is increasing. Despite this, the therapeutic options are limited and without a particularly strong evidence base. We present a rare, yet increasingly diagnosed, case of intralabyrinthine haemorrhage (ILH) together with radiological, audiological, and vestibular test results. Of note, this occurred spontaneously and has shown partial recovery in all the mentioned modalities.

## 1. Introduction

Sudden sensorineural hearing loss (SSNHL) was first described by de Kleyn in 1944 [1]. Today, it is defined as a sensori-neural hearing loss of >30 dB across at least 3 consecutive frequencies arising in <72 hours [2, 3]. It is generally unilateral and reports of its annual incidence vary from 5 to 300/100,000 [4, 5]. Up to 90% of cases are idiopathic [6] and treatment is generally with steroids, though the evidence supporting this is controversial [7–9] especially as spontaneous improvement occurs in >50% of patients within 2 weeks [6, 10, 11]. The specific type of steroid, its dose/duration, and method of delivery vary widely from unit to unit. Our unit uses very high dose oral dexamethasone and we have recently published encouraging results [12].

Intralabyrinthine haemorrhage (ILH) as a cause of SSNHL is extremely rare, and there have been few such reports in the literature [13–18]. The relationship of vestibular symptoms with spontaneous ILH is also unclear. Generally, haemorrhage occurs in patients with coagulopathies as seen in leukaemia or with anticoagulant medication and very few patients enjoy any recovery of function.

## 2. Case Report

A 50-year-old journalist presented to the ENT department at the University Hospital Zurich, with left-sided tinnitus, hearing loss, and general dizziness without real vertigo. The symptoms had suddenly appeared whilst at work a month previously and he had been treated by a private ENT doctor with oral prednisolone (50 mg daily for 10 days), betahistine, magnesium, and flunarizine with a diagnosis of an acute idiopathic vestibulocochlear loss. During this time his hearing loss had remained, but his dizziness symptoms had almost completely resolved. He also complained of tinnitus and a pressure sensation on the left ear. There was no further medical history of note other than that he took 100 mg aspirin daily because of borderline increased hematocrit. He occasionally drank alcohol and was an ex-smoker.

**Figure 1:**
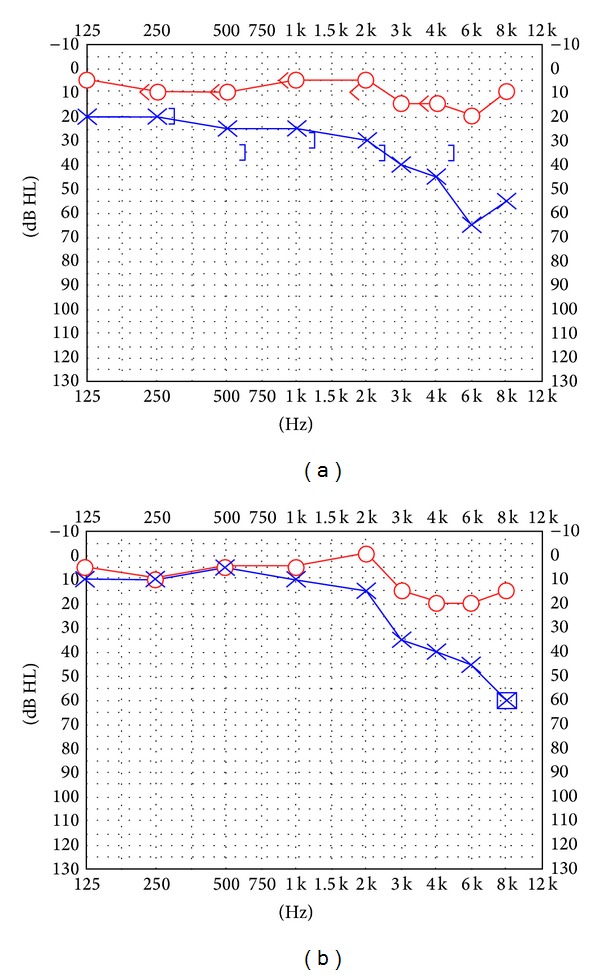
Pure tone audiogram at presentation (a) and at 3-month follow-up (b).

Otoscopy showed normal ear drums on both sides. There was no spontaneous or gaze evoked nystagmus. A clinical head impulse test was unclear as the patient continuously blinked. Head-shaking test showed nystagmus to the right. Pure tone audiogram (Figure 1) showed a clear asymmetry of his hearing.

**Figure 2:**
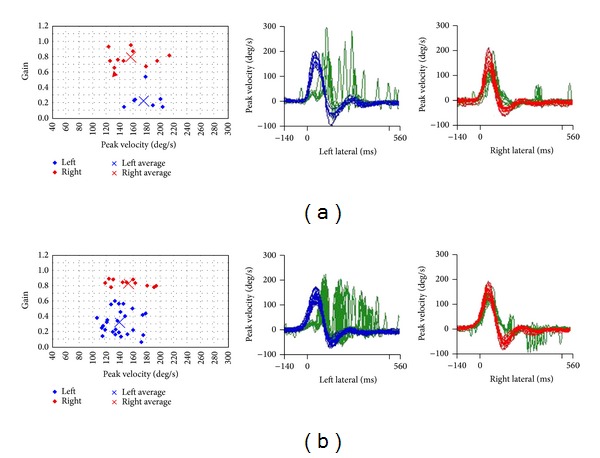
Video head impulse tests of the lateral semicircular canals at presentation (a) and at 3-month follow-up (b). Both show significant left sided correction saccades. DVA results however improved during follow-up.

The patient was referred for vestibular testing and an MRI. Video head impulse testing showed a complete loss of left-sided semicircular canal function (Figure 2) and a dynamic visual acuity (DVA) test was highly pathological for the left and marginally pathological for the right side. Sacculus testing with cVEMPs was borderline pathological on the left, and utriculus testing with oVEMPs showed a 100% loss on the left. Caloric testing showed a 77% asymmetry with feeble responses to cold water and no response to warm water.

**Figure 3:**
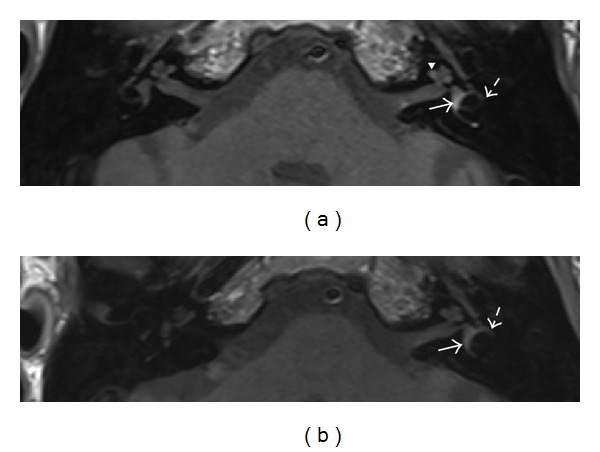
(a) T1 weighted native MRI of the petrous bone showing hemorrhage in the vestibule (arrow) and in the semicircular canals (partly shown, striped arrow) and slightly in the basal (not shown) and second turn (arrowhead) of the cochlea on the left. (b) 3-month follow-up shows clear resolution of the hemorrhage. Only slight residual blood is visible in the vestibulum (arrow) and in the semicircular canals (partly shown, striped arrow).

The MRI of the petrous bone (Figure 3) showed normal anatomy, but with high intensity signal on unenhanced T1 in both the cochlea and vestibulum, consistent with a left sided ILH.

The patient was asked to stop his aspirin and observed. Now 6 months after initial presentation his vestibular loss is well compensated and his DVA and video head impulse tests have improved, though they are still pathological. Furthermore his hearing has partially recovered in the lower frequencies (Figures 1 and 2). Follow-up MRI also shows resorption of the blood (Figure 3) with no residual in the cochlea but some in the vestibule.

## 3. Discussion

Overall, the vestibular sense is not well known by the general public (indeed, it would be the famous 6th sense) and patients often use a variety of terms when trying to describe their vestibular symptoms. This coupled with the fact that many patients experience excellent compensation in a matter of days to weeks means that a significant vestibular dysfunction may only be apparent with vestibular testing. These need not be with advanced equipment in specialized centres as simple bedside tests are reliable indicators of vestibular dysfunction [19]. Most reports in the literature only use caloric testing to evaluate vestibular function whereas today it is possible to examine all 5 components of the vestibular system, namely, the three semicircular canals, utriculus, and sacculus.

Hearing loss is a far better understood problem by the general public, and indeed our patient's main complaint was of this, combined with tinnitus. Tinnitus can sometimes be the only symptom of a high frequency hearing loss, and many patients are astonished to see how poorly they hear at high frequencies. The acuter and more asymmetrical the hearing loss the more noticeable for the patient and the more likely they are to present for investigation.

In situations of both cochlear and vestibular dysfunction, careful history taking and examination can often elucidate the cause. Important differentials include infectious, vascular [20, 21], traumatic, neoplastic [22], iatrogenic (surgical [23] or drug-related [21]), and neurological causes of dysfunction.

Controversy still exists however as to when a patient should be referred for imaging [24], and often a diagnosis of “idiopathic” hearing or vestibular loss is made, without imaging to rule out other causes. Clearly in cases of suspected stroke, imaging is mandatory, but, for example, our patient only was referred to hospital and indeed underwent imaging some weeks after his initial symptoms.

In MRI, the labyrinthine endolymph has essentially the same signal intensity as cerebral spinal fluid (CSF) in both T1 and T2 sequences. (Perilymph has almost the same electrolyte composition as CSF although, higher protein whereas endolymph has the same protein concentration but higher potassium and lower sodium [25].) T1 hyperintense signals can in theory result from fat, protein, or blood [23]. Fat in the labyrinth is only seen in very rare lipomas. Protein in the perilymphatic fluid can be seen in middle ear infections or bacterial labyrinthitis. Careful radiological examination, especially with the use of 3D-Flair sequences [26], can effectively differentiate between ILH, acute inflammation and breakdown of the blood-labyrinth barrier [27] without the need for histological confirmation. However, as yet, the prognostic or therapeutic relevance of these findings is unclear.

To date, nearly all cases reported in the literature have permanent dysfunction following ILH with MRI findings still apparent >6 months after the original presentation. Happily for our patient, we were able to document audiological, vestibular, and radiological improvements.

## 4. Conclusions

ILH is a rare diagnosis best made on MRI. Most reports in the literature are of ILH secondary to clotting disorders, and indeed our patient had been taking low dose aspirin. It may be however that the phenomenon is underreported due to underimaging of patients and difficulties in accurate radiological diagnosis [28]. Histological confirmation is however no longer required. Furthermore, due to its rarity there is little consensus as to the optimal therapy, though treatment directed at any underlying cause (e.g., reversal of anticoagulation) would seem intuitively plausible. Certainly, efforts should be made to uncover occult bleeding diatheses. Of note, steroids and hyperbaric oxygen have not been shown to be superior to allowing the condition to run its natural course.
